# The Challenge Initiative: Lessons on Rapid Scale-Up of Family Planning and Adolescent and Youth Sexual and Reproductive Health Services

**DOI:** 10.9745/GHSP-D-24-00153

**Published:** 2024-05-21

**Authors:** Clea Finkle, Yacine Bai, Venkatraman Chandra-Mouli, Samuel O’Keefe, Moses Tetui, Suzanne Fischer, Kojo Lokko, Lisa Mwaikambo, Saori Ohkubo

**Affiliations:** a Independent consultant, Seattle, OR, USA.; b Johns Hopkins Bloomberg School of Public Health, Baltimore, MD, USA.; c Formerly of the Department of Sexual and Reproductive Health and Research, World Health Organization, Geneva, Switzerland.; d University of Amsterdam, Amsterdam, Netherlands.; e School of Public Health Sciences, University of Waterloo, Waterloo, Canada.; f School of Pharmacy, University of Waterloo, Kitchener, Canada.; g Department of Epidemiology and Global Health, Umeå University, Umeå, Sweden.; h Independent consultant, Newland, NC, USA.; i William H. Gates Sr. Institute for Population and Reproductive Health, Baltimore, MD, USA.; j Johns Hopkins Center for Communication Programs, Baltimore, MD, USA.

## Abstract

The articles in this supplement showcase The Challenge Initiative’s strategies and insights for sustainably scaling evidence-based family planning and adolescent and youth sexual and reproductive health interventions and emphasize the need for multipronged interventions that address the complex web of factors influencing adolescents’ and youth’s access to contraceptive services.

## INTRODUCTION

Over the past several decades, the global health community has accumulated collective knowledge of what works to expand and improve family planning (FP) programming, including evidence-based interventions to shape the broader enabling environment, service delivery, and social and behavioral change, as well as new contraceptive formulations.^1^^,^^2^ However, these practices and commodities have often not spread through health systems in countries around the world.^3^^–^^5^ The challenges of coordinating stakeholders in and outside of government to effectively implement high-impact practices often confound leaders and managers at the local and community levels. Further, the global development community has typically invested in small, pilot-based, high-intensity programs whose design makes scaling to provide expanded and sustained coverage prohibitively costly.

Globally, by 2050, an estimated 7 of 10 people will likely live in urban areas, most in low- and middle-income countries (LMICs), where the pace of urbanization is projected to be the fastest. In this growing urban population, some of the poorest and least well-served are in informal settlements, which have typically lacked infrastructure and received few city resources.^6^ However, while the evidence is limited or mixed, the ongoing trend of political decentralization—shifting the decision-making authorities from the national government to the subnational governments—in LMICs could improve health system performance and outcomes in some settings.^7^^–^^9^ Helping cities and other subnational governments to achieve sustainable urbanization, and particularly to respond to the health needs of their most marginalized community members, is critical to successful development outcomes.

There is increasing global recognition of the human right to decide whether, when, and how many children to have. At the same time, investing in and scaling upaccess to contraception for women and girls—particularly married and unmarried individuals ages 15–24 years living in Africa and Asia—brings rich social, economic, educational, and environmental returns and proven reductions in women’s, children’s, and infant morbidity and mortality.^10^^,^^11^

To that end, The Challenge Initiative (TCI) partnered with local governments in various countries to take proven interventions and strategies to scale with local governments taking the lead by not only identifying the interventions that were most needed in their context but also committing human and financial resources from the beginning to enhance the likelihood of sustained impact. Funded by The Bill & Melinda Gates Foundation, Bayer AG, and Comic Relief, TCI covered 106 localities in 10 countries across 4 regions between 2016 and 2021.

The articles in this supplement provide important new evidence, collective experiences, good practices, and lessons learned about the strategies for helping local governments to sustainably scale access to FP for women, youth, and adolescents and to improve access to and use of contraception among adolescents and youth (AY). In this supplement, we present lessons for 2 broad audiences: those interested in the scale and sustainability of FP and other related health and development interventions and those interested in advancing understanding of scaling AY sexual and reproductive health (AYSRH) programming, with a particular focus on contraception.

## SCALING AND SUSTAINABILITY OF FAMILY PLANNING INTERVENTIONS

Finkle et al.^12^ cite many known challenges to the widespread adoption of FP interventions, including “underestimating the importance of politics and policy, failing to ensure local government ownership from the beginning, misjudging the time and resources needed for successful scale-up with sustainable impact, and the failure to adapt or make portable an innovation shown to be effective in one context to a new context.” The authors note that a growing body of literature on scaling has led to general agreement on the stages of scaling, the importance of adaptive management, the critical role of continuous engagement with local stakeholders, and the need to design for sustainability. However, the need to scale up health interventions for poor urban communities continues to be acute given the rapid pace of growth and gap in our understanding of how best to meet their needs, including for contraceptives, at scale in a sustainable way. The authors briefly summarize TCI’s experiences in and contributions to the field of scale-up theory and practice.

Ishola et al.^13^ describe a mixed-methods comparative case study to determine facilitators and barriers to subnational (state-level) adoption, implementation, and scale-up of high-impact FP/AYSRH interventions. The authors found that among 3 Nigerian states that had received substantive coaching support and financial investment, 2 states were higher performing and one lower performing, as determined by growth in FP client service volume over 24 months of implementation. One of the most important implications of this study is that political and managerial capacity—as opposed to just technical knowledge about FP—are most seen as linked with the successful adoption and scale-up of evidence-based FP/AYSRH interventions. This finding supports the emphasis on working with local governments to build their capacity to coordinate, finance, and implement already proven interventions.

Igharo et al.^14^ discuss the threat to development financing in Nigeria posed by declining donor funding, low government accountability, and weak governance. These problems were compounded for FP-related funding due to the limited understanding among policymakers of how investments contributed to the country’s broader health and development goals. These issues were addressed through an innovative, multipronged co-financing model, through which seed money was provided incrementally to Nigerian partner state governments as an incentive to commit and release state government funds for FP. Before the model was implemented, only 4 of 13 states had a dedicated budget line. By 2020, 10 of 13 partner states had budget lines and released funds for FP. The average amount of funds released per state more than tripled from 2018 to 2019, though decreased moderately in 2020. Over the engagement period (approximately 4 years), partner states were expected to increase their share of funds allocated to FP programs as TCI’s share gradually decreased. This intervention built on and strengthened existing state government financial processes and institutions toward increasing domestic financing, resource optimization and leverage, and fiscal accountability and transparency.

Kandji et al.^15^ describe how local municipalities and health systems in 5 countries in Francophone West Africa were supported to implement Family Planning Special Days (FPSDs) to reach new contraceptive users. Through FPSDs, municipalities provided free FP services on specific days in poor urban communities. The FPSDs’ management and tracking tools were the same as those already in use by the health system. The intervention strengthened collaboration among and ownership by municipalities and the health system and using existing health system tools strengthened sustainability.

### Collective Lessons Learned on Family Planning Interventions

These 4 articles collectively capture critical lessons in enhancing the scalability and sustainability of FP and related global development efforts.

- To ensure governments’ interest and sustainable engagement in addressing local FP priorities, the partnership emphasizes local ownership and investment and provides coaching to align interventions with local practices and policies.
- Effective advocacy proved to be critical in fostering sustained financing for health programs, building consensus in support of the model, and establishing a compelling case that the investment in FP is an investment in a country’s overall health and prosperity.
- Working through local governments’ and health systems’ existing structures, roles, and mechanisms (such as technical working groups, training and supervisory personnel, financial processes, and annual operation plans) to effectively transfer knowledge and skills and promote a culture of leading change enabled local governments to sustain various initiatives on their own.
- The involvement of strong champions external to local governments and health systems (such as religious and traditional leaders and other high-profile influencers) was essential for building public support for FP use and holding governments accountable for providing FP services.
- Using locally generated data (as opposed to conducting resource- and time-intensive population surveys)—backed by the commitment of local governments to improve data quality in the state/district-based HMIS—improved planning and decision-making, lowered the cost of monitoring and evaluation, and ultimately promoted sustainability.

## SCALING ADOLESCENT AND YOUTH SEXUAL AND REPRODUCTIVE HEALTH INTERVENTIONS

Globally, levels of contraceptive uptake have increased and unmet need for modern methods has decreased. Despite being slow and uneven in some places, similar trends can be observed for adolescents, whose levels of pregnancy and childbearing have decreased, attributable to increases in adolescent contraceptive uptake.^16^ One must appreciate these significant gains in AYSRH to forge ahead in a way that maintains the momentum gained in recent decades and that capitalizes on lessons learned. This must be done while not losing sight of various realities that require continued attention, including considerable regional disparities in modern contraceptive uptake among adolescents and, in some areas, only nominal improvements over time, despite their significance.^16^

Indeed, AY in LMICs still face significant challenges related to SRH. These include, among other risks, unintended pregnancies and unsafe abortions, which remain prevalent among this age group.^17^ These negative health outcomes affect adolescents by not allowing them to continue their education, pursue employment opportunities, and participate more fully in their communities, limiting their contribution to the overall development and prosperity of their families and communities.^18^^,^^19^ These outcomes also have broader social and economic implications, including limiting the potential to reap the benefits of a demographic dividend, including workforce growth and improved human capital.^20^

To mitigate these risks, we must prioritize AY’s access to and use of contraceptives and help them overcome the numerous barriers they face to reproductive health care. These obstacles include limited knowledge, stigma resulting from cultural norms and societal attitudes, lack of sexuality education, and provider bias.^21^ Both married and unmarried adolescents require contraceptive options, but they often struggle to obtain them and are sometimes denied access by providers.^22^ Other barriers would include the policy framework and laws of countries not explicitly supporting AYSRH.

In response, proven AY-focused interventions that are delivered effectively can help to ensure increased quality of tailored services, greater acceptance of the services among youth, and subsequently increased uptake of contraceptive services.^23^ The World Health Organization (WHO) provides official guidance and user-friendly materials to support the design and implementation of adolescent-friendly services, which remain comprehensive in scope.^24^ For example, the WHO Southeast Asia Regional Office’s supervisory checklist for adolescent-friendly services acknowledges the importance of ensuring that health care structures provide not only services but also information, education, and demand generation that include contraception and sexually transmitted infections.^25^ In this way, adolescent-friendly services, when delivered in a comprehensive way and aligned with WHO standards, can be effectively poised to address barriers to contraceptive uptake, adolescent pregnancy prevention, and sexually transmitted infections and HIV prevention. Therefore, to address the unique contraceptive needs and preferences of AY, comprehensive strategies appear fruitful for their ability to concurrently target and prevent adolescent pregnancy and other risks for their SRH and well-being.

### Adolescent and Youth Tailored Programming

The AY-focused articles in this supplement recognize the need for tailored programming that responds to the specific sensitivities and requirements of AY. Bwire et al.^26^ present a program strategy that supplemented general FP programming in Uganda by “layering” AYSRH services onto the structures and processes already implemented for women of reproductive age (WRA). The article details how program implementers paired facility-level changes with community-level sensitization activities to constitute a multipronged approach, addressing various systems that influence AY’s access to SRH information and services. Compellingly, the authors’ analysis of the program demonstrates how it not only was correlated with increased contraceptive uptake among AY in intervention sites but also augmented contraceptive uptake among WRA ages 25–49 years.

Two articles, Akila et al.^27^ and Avoce et al.,^28^ highlight mechanisms for improving AY-friendly services by engaging with existing FP entities, inclusive of existing planning and management units as well as health facilities, through supportive supervision, quality assessment, and consequent quality improvement processes. Akila et al. explain how supportive supervision and a cost-effective program monitoring tool can be harnessed to ensure compliance with essential guidelines for AY-friendly services, which include community engagement and demand generation activities. The article expands on TCI Nigeria’s development of a simplified checklist to guide supervisors and concurrent training of various health systems stakeholders in AY-friendly approaches. This case study suggests the feasibility and scalability of complementary quality improvement/quality assessment processes within existing monitoring systems and health infrastructures.

Similarly, Avoce et al. discuss an appraisal of the quality of adolescent-responsive contraceptive services in Zou, Benin. The process involved adapting a monitoring tool for adolescent- and youth-friendly health services and training an assessment team to carry out consecutive evaluations of health facilities and rank them through a simple classification system. This assessment, combined with a follow-up program to address gaps identified at the facility level, demonstrated improvements in services’ compliance with AY-responsiveness across several districts in Zou. In addition, the authors’ quantitative analyses show how differences in service quality between poor-ranking and high-ranking facilities (measured through the checklist and ranking system) were associated with significant differences in client volume, suggesting the validity of assessment tools and role of service quality in driving AY access to and uptake of contraceptive services. Both articles demonstrate the feasibility of adapting official AYFHS monitoring tools to local contexts and enacting them to inform program improvements.

Another key takeaway from the AYSRH articles is the importance of engaging the private sector to improve contraceptive use among adolescents. Akila et al.^29^ discuss how health care services and commodities have increasingly been accessed through proprietary patent medicine vendors (PPMVs) instead of primary health centers in Nigeria. There is a definite benefit in engaging the private sector; however, the authors note issues of quality in FP counseling and services, availability of long-acting methods, and integration of recordkeeping into the public health system and HMIS as key challenges that need to be addressed. Sharma et al.^30^ focus on how first-time parents (FTPs) are overlooked in state FP initiatives in India due to the lack of services adapted to the needs of adolescent and youth parents. The adolescent and youth FTP population in Uttar Pradesh presents a need for short-term and reversible contraceptives to promote spaced births. The article highlights the importance of engaging and coaching community health workers to provide FTP-specific counseling and outreach to meet the needs of this population.

Both articles demonstrate the successful use of additional health care stakeholders, such as private medical vendors and community health workers, to meet the targeted needs of AY and improve contraceptive use. The program in Nigeria strengthened the public-private partnership by establishing a functional referral system, government-led supportive supervision visits, and efficient data management. Using the TCI model, the authors were able to demonstrate the benefits of this public-private sector partnership between the PPMVs and primary health centers. Additionally, the results highlight the effect of leveraging PPMVs to provide AY with high-quality contraceptive information, services, and referrals to primary health centers. The program in India demonstrated how TCI’s coaching framework for engaging community health workers to provide targeted outreach services for FTPs yields more exposure to a wide range of contraceptive methods. Additionally, the results highlight a positive correlation between information exposure and contraceptive access among FTPs.

### Lessons Learned on Adolescent- and Youth-Friendly Services

The AY-focused articles in this supplement are unique because of the diversity of the regions and health systems they address, yet the authors also shed light on their similarities and lessons that might be more broadly applied.

The first is that integrating adolescent-responsive contraceptive services into FP services geared toward all WRA and their partners is feasible and can result in services that are sensitive to the needs and situations of AY, among other benefits. For example, harnessing preexisting supervision systems and public as well as private health care providers and entities prevents the need for creating parallel and often more costly systems and facilities and also helps contribute to the normalization rather than segmentation of AYSRH across health service structures. Consequently, AY can access services with greater discretion than if they were attending youth-only services, and youth’s trust in public health state services can grow over time as they transition to adulthood. These implications all make for a strong investment case for AY in resource-constrained settings because these integrated approaches are economically efficient and, as shown by Bwire et al.,^26^ can enhance contraceptive access among non-youth populations as well.

Second, the articles underscore the need for multipronged approaches to effectively address the complex web of systems that influence AY’s access to contraception and services. Hence, most of the AY interventions in this supplement simultaneously address demand generation and community engagement; social and behavior change among communities, providers, and youth themselves; and accessible information dissemination that reaches youth and various stakeholders and gatekeepers for AYSRH in communities.

Third, private sector outlets such as PPMVs are often AY’s trusted, preferred choice for health services. TCI’s interventions illustrate how engaging with the private sector—strengthening its quality of services, especially for AY, and linking their services to the public health system—can improve contraceptive use among AY.

## CONCLUSION

The articles in this supplement highlight promising lessons for sustainably scaling evidence-based health interventions. We believe the value of this supplement is its illustration of how the principles underpinning TCI—being demand-driven and centering local government leadership from the start, focusing on coaching instead of technical assistance, and emphasizing adaptive management approaches using near- to real-time data—show promising lessons for scaling and sustainability across a broad range of contexts and for health areas including and beyond those we specifically addressed.

Overall, the model was particularly successful in working with local governments and health systems to ensure political commitments and mobilize financial resources to provide quality FP services. From the collective lessons on AYSRH activities in particular, local decision-makers should also consider meaningful engagement with AY in designing and delivering interventions that serve them and advocate at subnational and national levels to ensure adolescent-responsive contraceptive services and comprehensive sexuality education.

In conclusion, we hope that the supplement will spark a conversation on how to sustain the impacts of these interventions and keep the needs of intended audiences, including AY, at the forefront of the global health agenda. The recommendations presented in this supplement should be considered in adapting and scaling FP and AYSRH interventions to other contexts, which will ultimately improve access and uptake of contraceptive services for all.

## Notes

**First Published Online:** XXXX.
